# The effects of neostigmine on postoperative cognitive function and inflammatory factors in elderly patients – a randomized trial

**DOI:** 10.1186/s12877-020-01793-4

**Published:** 2020-10-06

**Authors:** Bao Zhu, Defeng Sun, Lin Yang, Zhongliang Sun, Yan Feng, Chengcheng Deng

**Affiliations:** 1 Department of anesthesiology, The First Affiliated Hospital of Dalian Medical University, Dalian, 116011 China; 2 Department of Neuroelectrophysiology, The First Affiliated Hospital of Dalian Medical University, Dalian, 116011 China

**Keywords:** Neostigmine, Elderly patients, Cognitive impairment, Pro-inflammatory factors, Cholinergic anti-inflammatory pathway

## Abstract

**Background:**

Postoperative cognitive dysfunction is a common postoperative complication in elderly patients. In elderly patients, the decline of organ function and neuromuscular junction function make them more sensitive to muscle relaxants. They are more likely to experience residual muscle relaxation after surgery, which may cause various adverse events. Neostigmine, a commonly used muscle relaxant antagonist, can reduce the expression of inflammatory factors, thereby reducing the pro-inflammatory response and neurodegeneration of the cerebral cortex and hippocampus after surgery. The study aimed at observing the effect of different doses of neostigmine on postoperative cognitive function and peripheral inflammatory factors in elderly patients.

**Methods:**

One hundred thirty-two elderly patients who underwent a radical section of gastrointestinal cancer at First Affiliated Hospital of Dalian Medical University were divided into neostigmine and saline groups at a 2:1 ratio. Neostigmine was intravenously injected in the post-anesthesia care unit (PACU) according to the train-of-four ratio (TOFR) T4/T1. When TOFR was ≤0.5, 0.04 mg/kg neostigmine was administered, whereas when TOFR was > 0.5, 0.02 mg/kg neostigmine was injected. The main observation indexes were cognitive function, interleukin 1 beta (IL-1β), tumor necrosis factor-alpha (TNF-α), and interleukin 6 (IL-6) in peripheral blood at the different times before and after the surgery. Secondary observation indicators include the number of atropine injection, extubating time, PACU residence time, incidence of hypoxemia, hypercapnia, and postoperative nausea and vomiting in PACU, time of exhaustion, and length of hospitalization.

**Results:**

The extubating and PACU times in 0.04 mg/kg and 0.02 mg/kg groups were significantly shorter than those in the control group (*P* < 0.001). The incidence of early postoperative cognitive decline in 0.04 mg/kg and 0.02 mg/kg groups was 10 and 15.7%, respectively, which were significantly lower than those in the control group (*P* = 0.013).

**Conclusion:**

In elderly patients, 0.02–0.04 mg/kg neostigmine could significantly reduce the incidence of early postoperative cognitive decline without affecting peripheral inflammatory factors.

**Trial registration:**

Trial registration: Chinese Clinical Trial Registry, ChiCTR2000031739. Registered 8 April 2020 - Retrospectively registered, http://www.medresman.org.cn.

## Background

Postoperative cognitive dysfunction (POCD) is a common postoperative complication observed in elderly patients, which might cause insanity, anxiety, personality change, memory loss, significantly affects the recovery of patients. However, the exact mechanism of POCD in postoperative elderly patients is not yet clearly known; there were some hypotheses about POCD, such as neuroinflammation response and declined cholinergic system function. Currently, it is understood that the inflammatory response plays a vital role in the pathology of POCD.

Trauma during surgery increases the blood-brain barrier (BBB) permeability. Large-scale inflammatory factors (such as interleukin 1 beta (IL-1β), tumor necrosis factor-alpha (TNF-α), and interleukin 6 (IL-6)) owing to anesthesia and surgery are rapidly released in a short duration. This leads to the peripheral local inflammatory response. Furthermore, the inflammatory factors enter the central nervous system via the damaged BBB and activate the central immune-related cells to release a variety of inflammatory substances. These substances interfere with the neuronal activity and synaptic transmission, ultimately affecting the patient’s cognitive function [1, 2].

Due to the functional decline at the aging neuromuscular junction and the function reduction of various other organs, elderly patients are highly sensitive to muscle relaxants. Thus, they are likely to develop residual neuromuscular blockade (RNMB), which causes adverse reactions. Currently, neostigmine is commonly used clinically to antagonize postoperative RNMB. It can reversibly inhibit acetylcholinesterase in the active site through serine carboxylation, promote the impulse transmission between the neuromuscular junctions, and improve the cholinergic effects to antagonize the residual effect of muscle relaxants [3]. The cholinergic anti-inflammatory pathway proposed by Borovikova et al. is a neurohumoral mechanism that plays an important role in inhibiting the inflammatory response [4]. Treatment using acetylcholinesterase inhibitors enhance the transport of acetylcholine, which can potentially prevent neuroinflammation. Animal experiments have shown that the acetylcholinesterase inhibitor neostigmine could reduce the pro-inflammatory response and neurodegeneration of the postoperative cerebral cortex and hippocampus by reducing the activity and level of acetylcholinesterase [5].

The purpose of this study was to observe the effect of different neostigmine doses on the early postoperative cognitive function and peripheral inflammatory factor levels in elderly patients after laparoscopic surgery. Furthermore, the study also aimed at exploring the effective prevention methods for early postoperative cognitive decline in elderly patients.

## Methods

This study was approved by the Ethics Committee of the First Affiliated Hospital of Dalian Medical University (PJ-KY-2018-50(X)) and registered on the Chinese Clinical Trial Registry (Registration number: ChiCTR2000031739). Written informed consents were obtained from all the patients prior to their participation in the study.

### Participants

Patients involved in the study were those who were to undergo the radical section of gastrointestinal tumors at the First Affiliated Hospital of Dalian Medical University between September 2018 and January 2020. The inclusion criteria for the study were patients within 65 to 92 years of age, weighing between 40 and 93 kgs, having body mass index (BMI) between 16.8 and 33.3 kg/m^2^, with American Society of Anesthesiologists (ASA) Grade I–III, with Mini-Mental State Examination (MMSE) > 23, having an education level higher than a junior high school, with no contraindications of neostigmine, with no obvious infections or respiratory complications, with no liver and kidney dysfunctions, with no electrolytes or blood glucose abnormalities, with no severe visual and hearing impairments, with no history of drug and alcohol abuse, with no history of glaucoma or bromide allergy, with no history of neurological and psychiatric diseases or hyperthyroidism, and who had not taken sedatives, analgesics, or antidepressants within 1 month prior to the study. The exclusion criteria included postoperative infection [6], perioperative infusion of more than three units of red blood cells, and insufficient postoperative analgesia (Visual Analogue Scale (VAS) > 6 points).

### Sample size calculation

According to the preliminary experiments, the incidence of POCD in elderly patients undergoing different types of surgery was between 13 and 50%, with the incidence of POCD increasing with age [7]. The estimated effect size (f) of the neostigmine group and the control group was expected as 0.38. A two-sided test was arranged with a test efficiency value of 80% and a test level of 0.05. Using power analysis and sample size (PASS) 15.0 software, the sample size for each group was set to at least 36. Considering 10% of follow-up and withdrawal losses, the final total number of patients required for each group was 44, for a total of 132 patients.

### Grouping and administration of neostigmine

According to the order in which the patients entered the operating room, the Statistical analysis system (SAS) 9.4 software was used to randomize numbers 1 to 132 into the neostigmine group and the normal saline group according to 2:1. In PACU, neostigmine (1 mg/2 ml, Batch Number: 1810604, Shanghai Xinyi Jinzhu Pharmaceutical Co., Ltd.) was administered to the patients in the neostigmine group based on the TOFR by an anesthetic nurse. TOFR was monitored by TOF-GUARD INM type acceleration muscle relaxation tester (TOF-Watch SX, Organon, Ireland) immediately after the patients entered PACU. The contraction response of the adductor pollicis muscle was measured by stimulating the ulnar nerve through a transducer converter (TOF-Watch SX, Organon, Ireland). The parameters were set as TOF mode, current intensity 60 mA, with four series stimulations every 13 s. When the TOFR was ≤0.5, 0.04 mg/kg neostigmine was administered to the patients, whereas when it was > 0.5, 0.02 mg/kg neostigmine was administered [8–10]. The same volume of saline was administrated to the patients in the control group by the same anesthetic nurse, who recorded the group condition of each patient.

### Process of anesthesia administration

Prior to surgery, the patients were put on a 6-h fasting and 2-h water fasting period without any preoperative medication. Once the patient entered the operation room, the electrocardiogram, blood oxygen saturation (SpO_2_), and non-invasive blood pressure were monitored. Radial artery puncture and catheterization were performed for blood gas analysis and monitoring of the arterial blood pressure. Internal jugular vein puncture and catheterization were performed to monitor the central venous pressure. Furthermore, the bispectral index (BIS) and axillary temperature were also monitored.

All the patients underwent rapid intravenous induction via intravenous injection of 0.03 mg/kg midazolam, 0.2 μg/kg sufentanil, 1–2 mg/kg propofol, and 0.3 mg/kg cisatracurium besylate. Endotracheal intubation was performed using a visual laryngoscope. After intubation, the parameters of the anesthesia machine were set as tidal volume 8–10 ml/kg, respiratory rate 8–12 time/min, airway pressure < 30 mmHg, and partial pressure of carbon dioxide during expiration between 35 and 45 mmHg.

During the operation, a micropump was used for continuous intravenous infusion of 4 to 6 mg/kg/h propofol, 0.3 to 0.4 μg/kg/h sufentanil, and 0.15 to 0.20 mg/kg/h atracurium besylate. The intraoperative mean arterial pressure (MAP) fluctuation range was maintained below ±20% of the preoperative baseline value. When the MAP was below 20% of the baseline value, 1 mg/time methoxamine was administrated, whereas when it was above 20% of the baseline value, 5–10 mg urapidil was administered. When the heart rate was above 90 beats/minute, 1 mg/kg esmolol was administered, whereas when it was below 50 beats/minute, 0.5 mg atropine was administered. The intraoperative TOF count was maintained at 0. BIS was maintained between 40 and 60. The axillary temperature was maintained between 36.0 °C and 37.4 °C. The intraoperative intravenous solute was set at 6–8 ml/kg/h.

The intravenous injection of cisatracurium besylate was discontinued at the beginning of skin closure. When 30 min were remaining for the surgery to end, 0.5 to 1 mg butorphanol was injected intravenously, and an analgesic pump was connected for patient-controlled intravenous analgesia (PCIA) (8 to 12 mg butorphanol was added into 100 ml saline, infusion dose: 2 ml/hour, demand dose: 2 ml/time, lock time: 15 min [11]). All the patients were transferred to PACU with the intubation within 10 min after the surgery. The neostigmine was intravenously injected based on the TOF value. The patients in the control group were intravenously injected with an identical saline volume. The vital signs of the patients were closely observed in the PACU, and the arterial blood gas test was conducted prior to tracheal catheter removal. When the BIS value exceeded 90, and the modified Aldrete score was ≥12 points, the patients were sent to the ward.

### Cognitive function evaluation

The MMSE was used to assess the cognitive function of the patients. The test was performed 1 day before the operation (D0), and then on Day 1 (D1), Day 3 (D3), and Day 7 (D7) after the operation by the same psychiatrist. The total MMSE score was 30 points, and the difference between the postoperative and preoperative MMSE scores was ≥2 points, which suggested that the patient had a postoperative cognitive function decline [12].

### Inflammatory factors detection

Three millilitres of venous blood was collected 10 min before the induction (T1) while leaving the PACU (T2), Day 1 (T3) after the surgery, and Day 2 (T4) after the surgery. The blood samples were placed in an ethylenediaminetetraacetic acid (EDTA) anticoagulant tube and centrifuged within 15 min at 3000 rpm for 10 min. Furthermore, the supernatants were collected and stored in the refrigerator at − 80 °C for subsequent tests. The concentration of IL-1β (ELS-CYT022, Alisa Biotechnology), IL-6 (ELS-CYT030, Alisa Biotechnology), and TNF-α (ELS-CYT039, Alisa Biotechnology) in the peripheral patient blood were detected by enzyme-linked immunosorbent assay (ELISA). The kit’s minimum detection concentration was below 1.0 pg/ml. All the steps were performed in accordance with the manufacturer’s instructions.

### Other observation index

The observation index includes the number of atropine injection, extubating time (time from stopping intravenous infusion of the medication to the removal of the tracheal tube), the residence time in PACU, and incidence of hypoxemia, hypercapnia, and hypotension, the incidence of adverse reactions such as postoperative nausea and vomiting, and the length of hospital stay.

#### Blinding

The anesthetists who performed the perioperative management and blood collection were blinded to the group condition. Cognitive function evaluation was performed by a psychiatrist.

### Statistical analysis

Statistic Package for Social Science (SPSS) 21.0 software (Chicago, USA) was used for statistical analysis. Data in normal distribution were expressed as mean ± standard deviation (SD), and data that were not normally distributed were expressed as median (M) and quartile interval (IQR). Qualitative data were expressed in terms of frequency (rate). One-way analysis of variance and nonparametric test (rank kernel test) were used for comparing the differences between the three groups. Furthermore, a chi-square test was used to analyze the difference between these groups. When P was < 0.05, the difference was considered to be statistically significant.

## Results

**Fig. 1:**
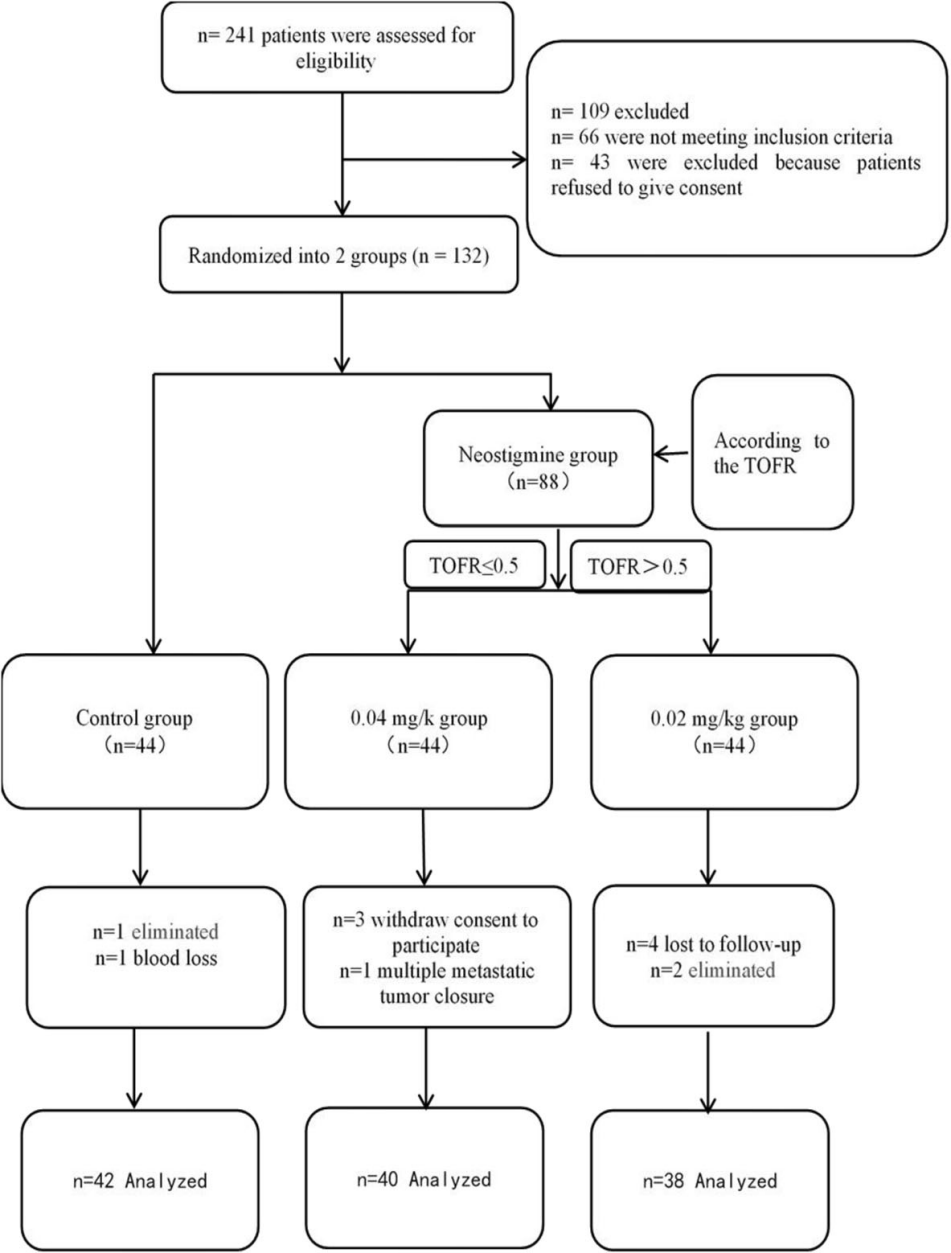
Flow chart of the study

In this study, 241 elderly patients who were scheduled for a radical section of gastrointestinal cancer were screened. Based on the inclusive and exclusive criteria, a total of 109 patients were excluded, 66 of whom did not meet the inclusion criteria, and 43 refused to participate in the study. Furthermore, 132 patients participated after signing the informed consent. During the study, owing to exclusion, rejection, and follow-up loss, 12 patients were excluded. Finally, data from 120 patients were analyzed. The flow chart is shown in Fig. 1.

### Basic features of the patients among the three groups

No significant differences were observed in terms of age, sex, BMI, preoperative MMSE score, ASA classification, type of surgery, and underlying diseases such as hypertension, coronary heart disease, arrhythmia, diabetes, stroke, anemia, and hypoproteinemia in the patients among these three groups (*P* > 0.05) (Table 1)

**Table 1:** Basic features of patients among the three groups

	Control group (*n* = 42)	0.04 mg/kg group (*n* = 40)	0.02 mg/kg group (*n* = 38)	*P-value*
Age (year, $\overline{\mathrm{x}}$±s)	72.9 ± 4.9	73.3 ± 6.2	72.5 ± 6.1	0.825
Sex (n,%)				
Male	22 (52)	21 (53)	20 (53)	0.751
Female	20 (48)	19 (47)	18 (47)	0.648
BMI (kg/m^2^, $\overline{\mathrm{x}}$±s)	23.5 ± 2.9	22.9 ± 2.7	22.7 ± 2.9	0.233
Preoperative MMSE score [M (IQ_R_)]	25 (23, 26)	25 (23, 27	25 (24, 27)	0.546
ASA grade (n,%)				0.643
I–II	21 (50)	24 (60)	21 (55.3)	
III	21 (50)	16 (40)	17 (44.7)	
Type of surgery (n,%)				0.735
Gastric cancer	23 (54.7)	19 (47.5)	15 (39.5)	
Colon cancer	19 (45.3)	21 (52.5)	23 (60.5)	
Diabetes (n,%)	6 (14)	8 (20)	5 (13)	0.136
Arrhythmia (n,%)	3 (7)	6 (15)	4 (10)	0.223
Stroke (n,%)	2 (5)	1 (2.5)	3 (7.9)	0.375
Anemia (n,%)	2 (5)	2 (5)	1 (2.6)	0.218
Hypertension (n,%)	15 (35.7)	14 (35)	13 (34)	0.093
Coronary heart disease (n,%)	3 (7)	4 (10)	5 (13)	0.835
Hypoproteinemia (n,%)	2 (5)	1 (2.5)	2 (5)	0.415

### Clinical features of the patients among the three groups during the operation

As listed in Table 2, the amount of midazolam, besylate, atracurium, sufentanil, and propofol during the operation, operation time, infusion volume, blood loss, and intraoperative blood transfusion remained similar among these three groups. There were no significant differences in the number of atropine injections (*P* > 0.05) and the incidence of adverse reactions such as hypoxemia and hypercapnia in PACU (*P* > 0.05). Compared to the control group, the extubating and PACU times in the 0.04 mg/kg and 0.02 mg/kg groups, respectively, were significantly reduced (*P* < 0.001), while there was no significant difference between the 0.04 mg/kg and 0.02 mg/kg groups (*P* > 0.05).

**Table 2:** Clinical features of patients among the three groups during the operation

	Control group (*n* = 42)	0.04 mg/kg group(*n* = 40)	0.02 mg/kg group(*n* = 38)	*P*-value
Midazolam (mg, $\overline{x}$±*s*)	1.9 ± 0.5	2.1 ± 0.6	2.0 ± 0.5	0.272
Atracurium, (mg, $\overline{x}$±*s*)	55 ± 30	63 ± 36	61 ± 37	0.694
Sufentanil (ug, $\overline{x}$±*s*)	74 ± 28	77 ± 30	75 ± 29	0.553
Propofol (mg, $\overline{x}$±*s*)	1540 ± 317	1770 ± 342	1632 ± 368	0.463
Infusion volume (ml, $\overline{x}$±*s*)	1558 ± 553	1515 ± 515	1678 ± 463	0.075
Blood loss volume (ml, $\overline{x}$±*s*)	180 ± 111	193 ± 98	201 ± 108	0.710
Blood transfusion volume (ml, $\overline{x}$±*s*)	28.9 ± 9.5	30.5 ± 7.0	25.7 ± 10.5	0.307
Operation time (min, $\overline{x}$±*s*)	189 ± 69	185 ± 71	188 ± 73	0.581
Extubating time (min, $\overline{x}$±*s*)	28.2 ± 9.6	21.4 ± 9.3^*^	21.5 ± 9.4^*^	0.000^*^
PACU time (min, $\overline{x}$±*s*)	63.5 ± 21.3	49.3 ± 10.7^*^	51.3 ± 11.5^*^	0.000^*^
Hypoxemia (*n*,%)	2 (5)	2 (5)	1 (2.6)	0.719
Hypercapnia (*n*,%)	2 (5)	1 (2.5)	2 (5.3)	0.835
Number of patients with atropine (*n*,%)	–	14 (32.5)	8 (21)	0.197

*P< 0.05 in comparison with the control group

### Comparison of the cognitive function of patients among the three groups

**Fig. 2:**
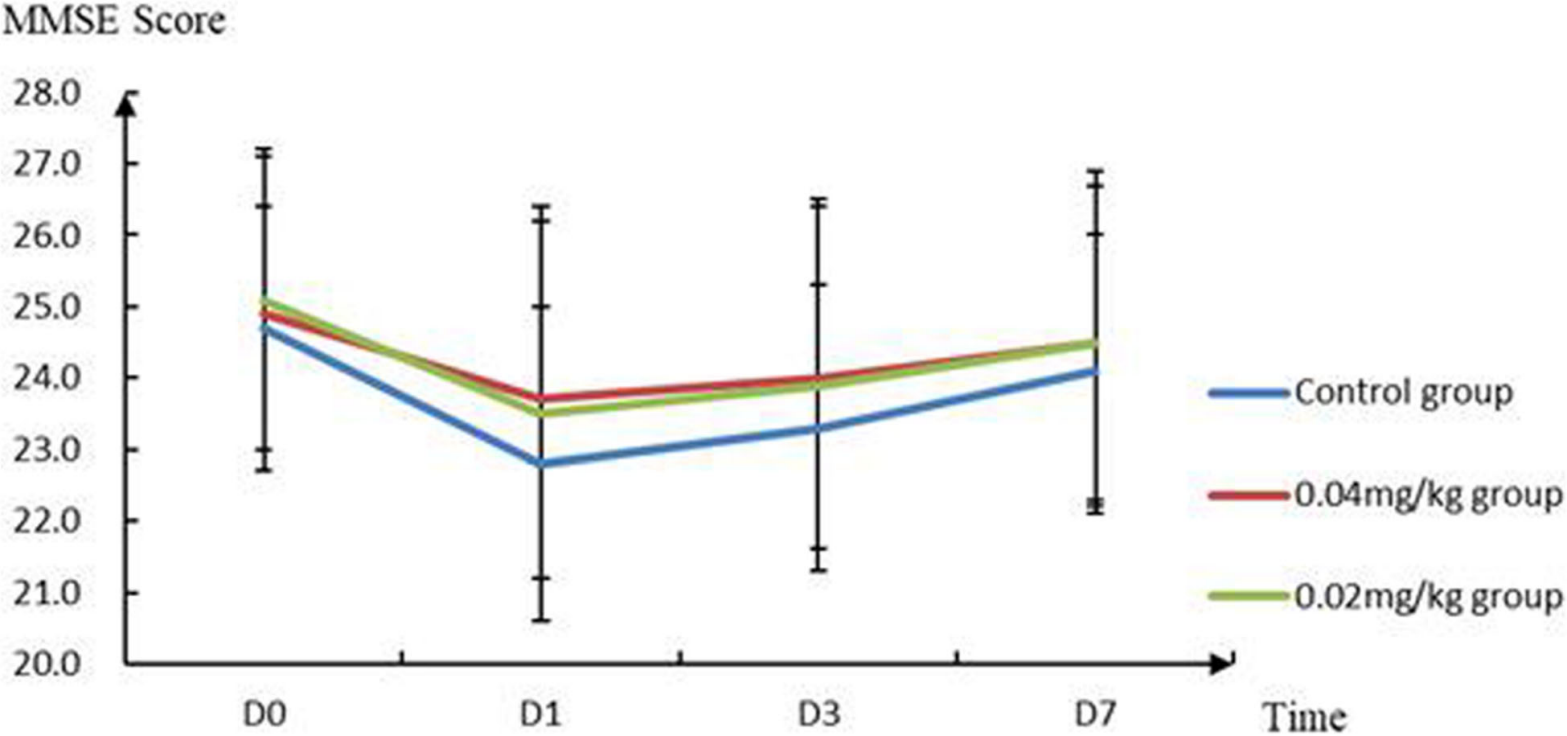
MMSE scores in the three groups at different times

As shown in Fig. 2, MMSE scores in the three groups at t2 are significantly lower than those at t1 (*P* < 0.05). Furthermore, MMSE scores at t3 and t4 are higher than those at t2 (*P* < 0.05). As listed in Table 3, the early postoperative cognitive decline rates in the 0.04 mg/kg and 0.02 mg/kg groups are 10 and 15.7%, respectively, which are significantly lower than those in the control group (*P* = 0.013). No significant difference was observed between the 0.04 mg/kg and 0.02 mg/kg groups (*P* > 0.05).

**Table 3:** Percentage of patients with declined cognitive functions among the three groups

	Control group (*n* = 42)	0.04 mg/kg group (*n* = 40)	0.02 mg/kg group (*n* = 38)	Neostigmine (*n* = 78)
Declined cognitive function (*n*)	14	4	6	10
Normal cognitive function (*n*)	28	36	32	68
Incidence (%)	33.3	10^a^	15.7^a^	12.8^a^

^a^*P*< 0.05 in comparison with the control group

### Comparison of inflammatory factor concentration in the three groups

**Fig. 3:**
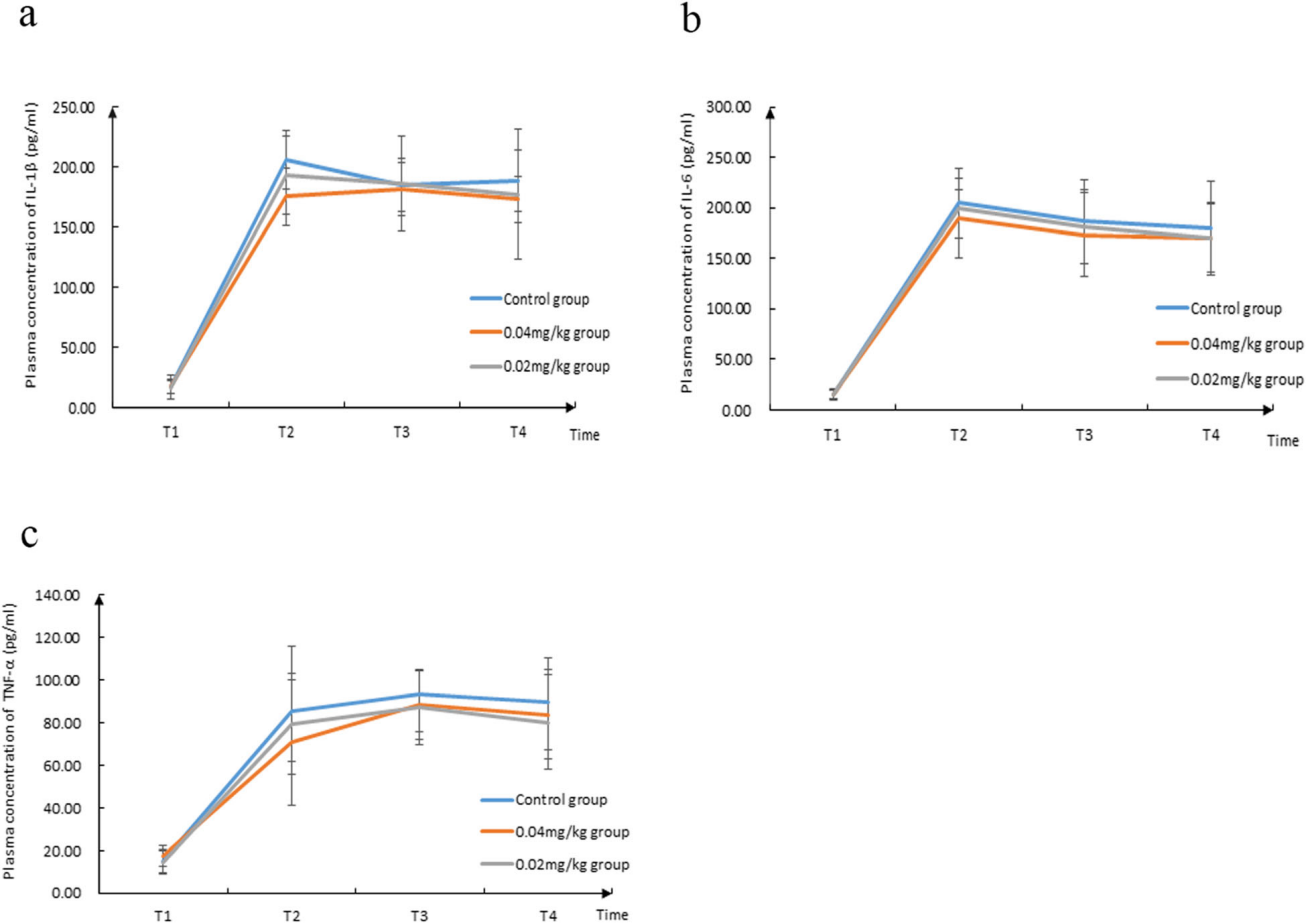
The concentrations of IL-1β, IL-6, and TNF-α in the peripheral blood among three groups at different times. **a** The concentration of IL-1β in the peripheral blood among three groups at different times. **b** The concentration of IL-6 in the peripheral blood among three groups at different times. **c** The concentration of TNF-α in the peripheral blood among three groups at different times

As shown in Fig. 3a–c, the concentrations of IL-1β, IL-6, and TNF-α inflammatory factors in the peripheral blood among three groups are significantly higher at T2, T3, and T4 compared to those at T1 (*P* < 0.05). In the case of T1, T2, T3, and T4, no significant difference was found between the three groups (*P* > 0.05).

### Comparison of postoperative data among the three groups

There was no significant difference in the postoperative pain VAS score, postoperative exhaust time, the incidence of postoperative nausea and vomiting (Postoperative nausea and vomiting, PONV), and hospital time among the three groups (*P* > 0.05), as listed in Table 4.

**Table 4:** Comparison of postoperative data

	Control group (*n* = 42)	0.04 mg/kg group (*n* = 40)	0.02 mg/kg group (*n* = 38)	*P* value
VAS score [*M (IQ*_*R*_*)*]				
1 h	2 (1,5)	2 (1,5)	3 (1,6)	0.316
24 h	3 (1,6)	3 (1,5)	2 (1,5)	0.294
48 h	2 (1,4)	2 (1,3)	2 (1,3)	0.471
Postoperative exhaust time (day, $\overline{x}$±*s*)	4 (2,7)	3 (2,5)	3 (2,6)	0.533
PONV (*n*,%)	4 (10)	4 (10)	3 (7.8)	0.304
Hospital time (day, *M* (*IQR*))	15 (7,45)	16 (8,34)	15 (8,46)	0.618

## Discussion

According to Fuchs-Buder et al., when inhaled anesthesia or intravenous anesthesia is used to maintain the partial neuromuscular block-level (TOFR > 0.4), small doses of neostigmine could quickly achieve active antagonism [8, 9]. In terms of recovery, 0.02 mg/kg of neostigmine takes 10 min, and 0.03 mg/kg of neostigmine takes 5 min. Chen Yanjun et al. proposed that 0.04 mg/kg of neostigmine can lower the retention time in the PACU in elderly patients without increasing the length of hospital stay [13]. Butterly et al. reported that patients with residual muscle relaxants stay in the PACU significantly longer compared to the ones without them [14]. The above literature suggests that applying 0.02–0.04 mg/kg of neostigmine to antagonize the residual muscle relaxation after the surgery can significantly reduce the patient’s recovery time. In this study, the extubation and PACU times of the patients in the 0.04 mg/kg and 0.02 mg/kg groups are significantly shorter than the control group (*P* < 0.001). This suggests that the muscle relaxant antagonist neostigmine can accelerate patient recovery, which is consistent with the conclusions of previous studies.

When the TOF value is lower than 0.9, the patients in PACU should be routinely monitored and given muscle relaxant antagonists to antagonize the residual muscle relaxation. Furthermore, when TOF returns to 0.9, owing to the high sensitivity of throat muscles to muscle relaxants, a risk exists of incomplete recovery of the throat muscle functionality. Some patients would suffer from adverse reactions such as dysphagia, drooping eyelids, and blurred vision [15]. Therefore, it is essential to determine the antagonistic time and dose of muscle relaxants [16]. In this study, based on TOFR, the patients in the neostigmine group were offended owing to the different treatments of neostigmine.

The maximum effective dose of neostigmine is 0.07 mg/kg. When the dosage is increased, the antagonistic effect is improved along with adverse reactions such as nausea, vomiting, and ataxia [17]. In this study, patients in PACU were monitored for neuromuscular recovery to obtain a significant evaluation of muscle relaxation recovery. However, no increase was observed in the incidence of adverse reactions such as postoperative nausea and vomiting as mentioned in the literature.

Zhang et al. reported that the postoperative cognitive function of patients showed a decline, followed by an increase [18]. The patients’ cognitive function scores were low 1 day after surgery, while the postoperative cognitive function gradually recovered. In this study, the MMSE scores at t2 of the three groups were significantly lower than those at t1, and the MMSE scores at t3 and t4 were higher than those at t2 (*P* < 0.05). This suggested that the early postoperative cognitive function of elderly patients is indeed reversible and recoverable with time. The incidence of early postoperative cognitive decline in the 0.04 mg/kg and 0.02 mg/kg groups was 10 and 15.7%, respectively, which were significantly lower than those in the control group (*P* = 0.013). No significant difference was observed between the neostigmine groups (*P* > 0.05), which indicated that the use of 0.02–0.04 mg/kg of neostigmine after the surgery could significantly reduce the incidence of early postoperative cognitive decline in elderly patients.

We found that the cognitive function of the three groups 1 day after surgery is significantly lower than that of 1 day before the operation. The concentrations of three inflammatory factors in the peripheral blood in the three groups at T2, T3, and T4 are significantly higher than T1. This suggests that surgical trauma can also cause the peripheral pro-inflammatory cytokine levels to rise while causing postoperative cognitive decline. Continuous monitoring of inflammatory factor changes in patients revealed that the concentration of inflammatory factors did not show any significant decrease within 2 days after the operation, which also suggested that the postoperative cognitive decline may be closely related to the continuous high inflammatory response concentration. The previous literature showed that cognitive dysfunction might last for days, weeks, several months, or beyond. Therefore, in 2018, few scholars suggested the use of perioperative neurocognitive disorder (PND) instead of POCD to describe the cognitive functional changes in patients before and 1 year after the surgery. The follow-up time is quite short, which is 7 days after surgery. Therefore, extended follow-up time is the focus of our future research, which is the reason why blood collection time is not consistent with the evaluation time.

According to certain studies, anesthesia and surgery activate the innate immune system, which is manifested by increased levels of pro-inflammatory cytokines. Furthermore, evidence exists that show how neuroinflammation plays a crucial role in the pathogenesis of POCD, and surgical trauma can induce the systemic inflammatory response and release various inflammatory factors. These inflammatory factors enter the central nervous system from the periphery via the damaged BBB and further activate microglia cells to secrete the corresponding cytokines. This, in turn, interferes with the neuronal activity and synaptic transmission, affecting the patients’ cognitive function. These effects are mainly caused by pro-inflammatory cytokines, such as IL-1β, TNF-α, and IL-6 [19–21]. Our study determines that the concentrations of IL-1β, IL-6, and TNF-α inflammatory factors in the peripheral blood of the three groups at T2, T3, and T4 are higher than those at T1 (*P* < 0.05), indicating that anesthesia and surgery lead to increased levels of pro-inflammatory cytokines, which is consistent with the literature results.

However, this study also determined that there are no significant differences in the concentrations of IL-1β, IL-6, and TNF-α inflammatory factors in the peripheral blood of patients in the three groups at T1, T2, T3, and T4 (*P* > 0.05). This suggests that neostigmine significantly reduces the incidence of early postoperative cognitive decline in elderly patients, and may not be involved with the peripheral inflammatory factor levels. A previous study showed that the hypofunction of the cholinergic system was related to cognitive dysfunction caused by neurodegenerative diseases such as Alzheimer’s disease, which was characterized by a gradual decline in cognitive function and hypofunction of the cholinergic system [22]. Acetylcholinesterase inhibitors prevented acetylcholinesterase from breaking down acetylcholine, thereby increasing the level and duration of the neurotransmitter acetylcholine. Certain cholinesterase inhibitors that could improve the memory function of patients with Alzheimer’s disease, such as donepezil and galantamine, could block the decomposition of acetylcholine by inhibiting acetylcholinesterase and enhance cholinergic transmission. It could alleviate the central inflammatory response in patients and relieve clinical symptoms, which could be used as a potential treatment to prevent neuroinflammation [23]. The trauma caused by anesthesia and surgery may increase BBB permeability. Neostigmine acted on the central nervous system via the damaged BBB and reduces acetylcholine hydrolysis by inhibiting cholinesterase activity from increasing the utilization of acetylcholine, which reduced the incidence of postoperative cognitive decline in patients. However, as this study did not monitor the changes of neurotransmitters and inflammatory factors in the central nervous system of elderly patients, it could not be clearly stated whether neostigmine played a role in the central nervous system or not.

The study has certain limitations. The follow-up time is short. Only MMSE is used to evaluate the patients’ cognitive function. In the future, additional relevant professional scales should be added to assess patients’ cognitive function. As only a few single types of surgeries have been performed, the results and conclusions of this study need further analyses and larger clinical data for verification.

## Conclusions

In summary, it can be said that the use of 0.02–0.04 mg/kg muscle relaxant antagonist neostigmine after surgery can significantly reduce the incidence of early postoperative cognitive decline in elderly patients, and may not play a role through peripheral inflammatory factor levels.

## Data Availability

The datasets used and/or analyzed during the current study are available from the corresponding author on reasonable request.

## Abbreviations

PACU: Post-anesthesia Care Unit
TOFR: Train-of-Four Ratio
IL-1β: Interleukin 1 beta
TNF-α: Tumor Necrosis Factor-alpha
IL-6: Interleukin 6
POCD: Postoperative Cognitive Dysfunction
BBB: Blood-Brain Barrier
RNMB: Residual Neuromuscular Blockade
BMI: Body Mass Index
ASA: American Society of Anesthesiologists
MMSE: Mini-Mental State Examination
VAS: Visual Analogue Scale
PASS: Power Analysis, and Sample Size
SAS: Statistical Analysis System
BIS: Bispectral Index
MAP: Mean Arterial Pressure
PCIA: Patient-controlled Intravenous Analgesia
ELISA: Enzyme-linked Immunosorbent Assay
SPSS: Statistic Package for Social Science
PND: Perioperative Neurocognitive Disorder
